# Cytotoxic T-Lymphocyte-Associated Protein 4 Deficiency Colitis Masked by Recurrent Cytomegalovirus Colitis Successfully Treated With Vedolizumab

**DOI:** 10.14309/crj.0000000000002225

**Published:** 2026-07-03

**Authors:** Roney Shibu, Gabin Soosaipillai, Sooraj Rajendran Pillai, Waled Mohsen

**Affiliations:** 1 Royal Brisbane & Women's Hospital, Brisbane, Queensland, Australia; 2 School of Medicine, University of Queensland, Brisbane, Queensland, Australia; 3 Pathology Queensland, Gold Coast University Hospital, Gold Coast, Queensland, Australia; 4 Department of Digestive Diseases, Gold Coast University Hospital, Gold Coast, Queensland, Australia

**Keywords:** cytotoxic T‐lymphocyte associated protein 4 deficiency colitis, vedolizumab

## Abstract

Cytotoxic T-lymphocyte-associated protein 4 (CTLA-4) deficiency is a primary immunodeficiency syndrome caused by mutations in the CTLA-4 gene. Its heterogeneous presentation, often with multisystem involvement, can result in delayed diagnosis and treatment. CTLA-4 deficiency colitis (CDC) is endoscopically and histologically indistinguishable from inflammatory bowel disease and may be masked by a secondary process, underscoring the importance of genetic testing. We present a case of corticosteroid-refractory CDC in a frail, comorbid woman that was masked by recurrent cytomegalovirus colitis. Vedolizumab was commenced and steroid-free clinical remission was achieved after 5 months. There was no recurrence of colitis until vedolizumab was self-ceased after 12 months. CDC flared 7 months post vedolizumab cessation but due to patient preference, it was not recommenced.

## INTRODUCTION

Cytotoxic T-lymphocyte-associated protein 4 (CTLA-4) is an inhibitory receptor on T cells that regulates T-cell-mediated responses to maintain immune homeostasis.^1^ Heterozygous germline mutations in the CTLA-4 gene result in CTLA-4 deficiency, a primary immunodeficiency characterized by infections, lymphoproliferation and multisystem autoimmune disease.^2^ Autoimmune manifestations are similar to those seen in antibody-mediated CTLA-4 blockade (like ipilimumab), including cytopenias, enterocolitis, psoriasis, and thyroiditis. CTLA-4 deficiency is inherited in an autosomal dominant pattern with incomplete penetrance (67%) and has a median age of onset of 11 years.^3^ Gastrointestinal manifestations include colitis, celiac disease, and autoimmune enteropathy. CTLA-4 deficiency colitis (CDC) can mimic inflammatory bowel disease (IBD), but this distinction is critical as it is often refractory to IBD therapies and responsive to targeted therapies like abatacept, a CTLA-4 immunoglobulin fusion molecule. Vedolizumab is an α4β7 integrin inhibitor used to treat moderate-to-severe IBD and immune checkpoint inhibitor (ICI)- induced colitis, with a favorable safety profile due to its gut-selective mechanism of action. We present a case of CTLA-4 deficiency presenting in late adulthood with classical Hodgkin lymphoma, cytopenias, and corticosteroid refractory autoinflammatory colitis masked by recurrent cytomegalovirus (CMV) colitis successfully treated with vedolizumab.

## CASE REPORT

A 61-year-old woman receiving adriamycin, bleomycin, vinblastine, and dacarbazine for Stage 4B mixed cellularity classical Hodgkin lymphoma was admitted with non-bloody diarrhea. Medical history was significant for breast cancer treated with lumpectomy, hydatidiform mole, multiple excised skin cancers and osteoporosis. There was a family history of Crohn's disease and CTLA-4 haploinsufficiency (daughter).

Physical examination revealed tenderness in the left iliac fossa without features of peritonitis and laboratory investigations showed a hemoglobin of 122 g/L (reference range [RR] 115–160 g/L), white blood cell count of 0.2 × 10^9^/L (RR 4–11 × 10^9^/L), neutrophils of 0.01 × 10^9^/L (RR 2–8 × 10^9^/L), platelets of 45 × 10^9^/L (RR 150–450 ×10^9^/L), and albumin of 33 g/L (RR 35–50 g/L). Norovirus stool PCR was positive and computed tomography (CT) showed mucosal hyperenhancement and wall thickening of the descending and rectosigmoid colon, consistent with colitis. Neutropenic infectious colitis was treated with intravenous (IV) augmentin, valaciclovir, and filgrastim. Bowel frequency improved to once daily on discharge.

Adriamycin, bleomycin, vinblastine, and dacarbazine was changed to mini-CHOP due to intolerance, and a complete metabolic response was achieved after 4 cycles. However, abdominal pain and diarrhea recurred during treatment (10 bowel motions daily), which was managed as chemotherapy-related diarrhea.

**Figure 1.**
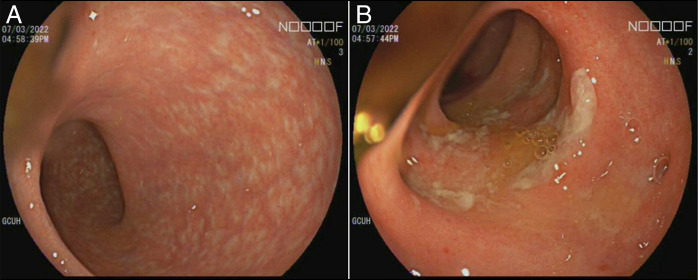
Patchy inflammation characterized by mild erythema and decreased vascular pattern [Mayo endoscopic score 1] in the rectum (A) and sigmoid colon (B).

**Figure 2.**
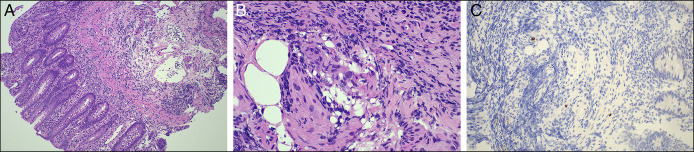
(A) H&E (10×) of sigmoid colon mucosa with increased numbers of inflammatory cells expanding the lamina propria without significant distortion of crypt architecture. (B) H&E (40×) of sigmoid colon showing rare enlarged atypical appearing cells were identified. (C) Cytomegalovirus (20×) immunohistochemistry shows positive nuclear reaction in atypical cells. H&E, hematoxylin and eosin.

Due to its prolonged course of 4 months, including 1 month post-chemotherapy and associated 5kg weight loss to 39 kg (body mass index 14.2), further investigations were performed. CT showed proctocolitis involving the rectosigmoid, descending, and ascending colon. Sigmoidoscopy revealed rectosigmoid erythema (Figure 1), and histological examination of the sigmoid colon showed ulceration and expansion of the lamina propria by a mixed inflammatory infiltrate comprising plasma cells, histiocytes, and neutrophils. Rare, enlarged, atypical appearing cells were identified, and CMV immunohistochemistry showed strong positive nuclear staining in these cells (Figure 2), confirming CMV inclusions. The patient had an excellent clinical and biochemical response to 5 days of IV ganciclovir and regained 2 kg over the next 2 months. But CMV colitis recurred after 3 months, requiring further IV ganciclovir (6 days) and a prolonged course of valganciclovir (3 weeks) on discharge. CT findings were unchanged and celiac serology, fecal elastase and fecal antitrypsin were normal. Esophagogastroduodenoscopy showed mild chronic gastritis with no evidence of *Helicobacter*-like organisms, atypical cytomegalic cells, or intranuclear viral inclusions. Bowel frequency returned to post-chemotherapy baseline of 3 semi-formed motions daily.

**Figure 3.**
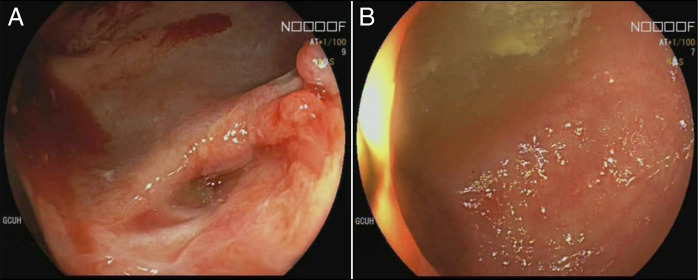
Inflammation characterized by loss of vascular pattern and erythema [Mayo endoscopic score 2] in the rectum (A) and sigmoid colon (B).

**Figure 4.**
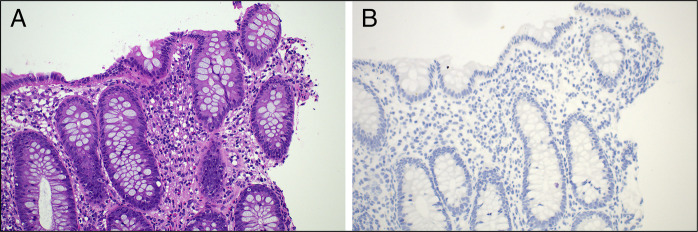
(A) Hematoxylin and eosin (20×) of colonic mucosa showing increased inflammatory cells within the lamina propria. No cytomegalic cells or definite viral cytopathic change was identified. (B) Cytomegalovirus immunohistochemistry (20×) showing negative reaction.

Diarrhea recurred after 6 months (10 loose bowel motions daily), but investigations for other etiologies, including infectious causes, were negative. Fecal calprotectin and C-reactive protein (CRP) were 1,400µg/g and 53 mg/L respectively. Sigmoidoscopy macroscopically showed worsening of rectosigmoid inflammation (Figure 3), and histology showed an active colitis without chronic inflammatory changes or histological features of IBD such as crypt architecture distortion, basal cell plasmacytosis, and granulomata (Figure 4). No viral inclusions or cytomegalic cells were identified, and CMV immunohistochemistry was negative. Magnetic resonance imaging of the pelvis demonstrated features of chronic proctocolitis without fistulas. This constellation of pancytopenia, malignancies, persistent colitis in identical distribution with incomplete response to antivirals and family history of CTLA-4 haploinsufficiency raised suspicion for underlying CDC. Whole exome sequencing was performed, which returned months later revealing a pathogenic CTLA-4 variant (NM_005214.5(CTLA4)c.60G>A, p.(Trp20*)), confirming the diagnosis.

**Figure 5.**
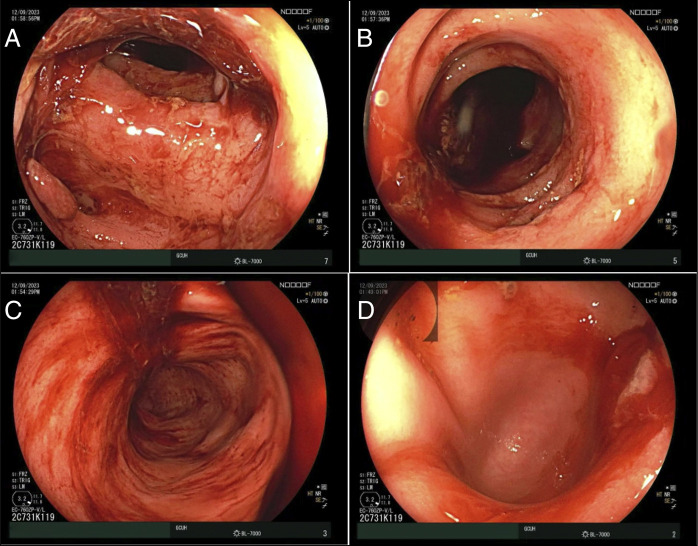
Large ulcers in the sigmoid (A) and descending (B) colon along with aphthous ulcers in the transverse colon (C) and cecum (D). NB: Significant bleeding from barotrauma on the right colon due to friable mucosa.

**Figure 6.**
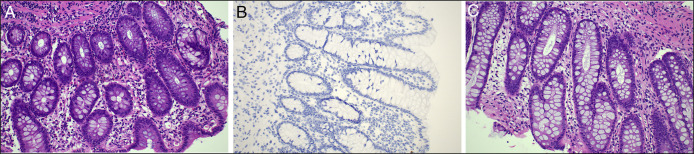
(A) H&E (20×) of colonic mucosa showing mildly increased inflammatory cells within the lamina propria with preserved crypt architecture. No viral inclusions, cytopathic changes or atypical cytomegalic cells were identified. (B) Cytomegalovirus immunohistochemistry (20×) showing negative reaction. (C) H&E (20×) of rectal mucosa showing preserved crypt architecture with no active inflammation, viral cytopathic change or atypical cytomegalic cells. H&E, hematoxylin and eosin.

Given her clinical frailty, osteoporosis and recurrent malignancies, upfront IV vedolizumab (300 mg at 0, 2, and 6 weeks) alongside 40 mg of prednisolone was commenced. Four-weekly vedolizumab was continued, and prednisolone was weaned. There was no significant clinical or biochemical response (CRP 51 mg/L and fecal calprotectin 970 µg/g) at 1-month follow-up. After 3 months, diarrhea settled to baseline 1–3 semi-formed bowel motions and fecal urgency resolved, but she remained on 15 mg of prednisolone, suggesting partial response. CRP and fecal calprotectin improved to 29 mg/L and 610 µg/g respectively. Colonoscopy showed diffuse ulceration of the colon, worse in the left colon but with rectal sparing (Figure 5), and histology confirmed active colitis (Figure 6). Steroid-free clinical remission was achieved after 5 months. Esophagogastroduodenoscopy performed 1 month later for variceal evaluation in the setting of new cirrhosis showed CMV gastritis. However, she refused treatment.

The patient self-ceased vedolizumab 12 months after commencement but remained in clinical remission at 5-month follow-up. CRP and fecal calprotectin were 18 mg/L and 530 µg/g respectively. Considering the patient's preference to remain off treatment despite not achieving deep remission, vedolizumab was not restarted. She was admitted for concomitant CMV and *Clostridioides difficile *colitis 1 month later, when colonoscopy showed severe acute pancolitis. This was followed by a CDC flare 2 months later, confirming poor disease control off vedolizumab. But with guarded prognosis from rapidly progressing facial squamous cell carcinoma, a comfort-focused approach was pursued, and advanced therapies were not considered. She died 3 months later.

## DISCUSSION

Monogenic IBD like CDC presents a diagnostic dilemma due to the absence of pathognomonic clinical, endoscopic, or histological features. Guidelines suggest early-onset IBD, extensive extraintestinal manifestations, recurrent infections, multisystem autoimmune diseases, and positive family history should prompt early genetic testing^4^ but in practice, diagnosis is often delayed. Hypogammaglobulinemia and IBD refractory to traditional therapies like anti-tumor necrosis factor (TNF)-α inhibitors should also raise suspicion of underlying CTLA-4 deficiency. Recent studies have discovered microbiome and metabolomic signatures in CTLA-4 deficient patients, which may aid diagnosis in future practise.^1^ Development of validated risk stratification tools would enable cost-effective genetic testing of the high-risk population, potentially leading to earlier diagnosis.

Management of CTLA-4 deficiency requires consideration of organ manifestations and patient comorbidities. Treatment may include targeted therapies, immunoglobulin replacement and hematopoietic stem cell transplant in refractory cases. Targeted therapies for gastrointestinal manifestations include abatacept, sirolimus, anti-TNF-α and corticosteroids.^5^ Consensus guidelines for CDC are lacking, but the widely accepted treatment paradigm involves first-line corticosteroids followed by targeted therapies.

Vedolizumab is a humanized monoclonal antibody that inhibits α4β7 integrin expressed on gut-homing T-lymphocytes, thereby blocking lymphocyte trafficking into the intestinal mucosa. Its therapeutic role in non-IBD indications is expanding. Although it is approved for steroid-refractory ICI colitis, this is only the second case reporting its efficacy in treating CDC.^6–8^ CDC shares a pathophysiology similar to that of anti-CTLA-4 ICI colitis. In healthy individuals, CTLA-4 is highly expressed on regulatory T cells (T_Reg_), resulting in suppression of effector T cells (T_Eff_) and maintenance of self-tolerance.^9^ T_Reg_ function is impaired in CTLA-4 deficiency resulting in unchecked T_Eff_ activation. A recent study suggested that vedolizumab binds to T_Reg_ and T_Eff_ cells in a dose-dependent manner.^9^ It suggested vedolizumab preferentially inhibits T_Eff_ cell gut trafficking at low-moderate concentrations, allowing some residual T_Reg_ gut homing, which may partly restore immune homeostasis and contribute to its clinical efficacy. At very high concentrations, there was no significant difference in inhibition between T_Reg_ and T_Eff_. Post hoc analysis of GEMINI II and III trials also showed a non-linear exposure-efficacy correlation with higher remission rates in the 40–55 µg/mL vedolizumab group compared with the >55 µg/mL group.^9^ These mechanisms may explain vedolizumab's efficacy in CDC.

Vedolizumab's gut-selective mechanism of action offers a superior safety profile compared with systemic immunosuppression with infliximab and abatacept, particularly in frail patients. As it also does not increase the risk of malignancy, it was an excellent choice for our patient.^10^ Although further evidence of endoscopic healing and deep remission are needed, these properties make vedolizumab an attractive option in comorbid patients with CDC.

Abatacept (CTLA-4-Ig) is a selective costimulation modulator that mimics the function of CTLA-4 to inhibit T-cell activation. Case reports have reported its effectiveness in treating multisystem and refractory autoimmune manifestations of CTLA-4 deficiency including neurological autoimmunity, cytopenias, and colitis.^11–13^ However, abatacept increases risk of severe infections and its commencement requires molecular confirmation, which may take several weeks and limits its early use.

CMV is an immunopathogenic virus that can reactivate in immunocompromised hosts. Its role in IBD is poorly understood but several studies^14–16^ suggest that colonic CMV reactivation is associated with higher risk of steroid-refractory IBD. Although CDC is distinct from IBD, CMV has also been detected more commonly in refractory immunotherapy-related colitis.^17–19^ The potential role of CMV in intestinal immune dysregulation requires further investigation. Management of CMV colitis with concomitant autoimmune colitis may require antivirals and modulation of immunosuppression or inflammation. While corticosteroids favor CMV reactivation, vedolizumab is not associated with an increased risk of CMV colitis, thereby conferring a therapeutic advantage in this setting.^17,20^

## DISCLOSURES

Author contributions: W. Mohsen devised the project, identified key discussion points and R. Shibu drafted the manuscript. G. Soosaipillai and S. Pillai sourced and annotated the histology slides and contributed to discussion, particularly from a histopathological perspective. All authors critically revised the manuscript, approved the final version for submission and agree to be accountable for all aspects of the work. R. Shibu is the article guarantor.

Acknowledgments: We acknowledge the Gold Coast University Hospital histopathology department for sourcing and photographing the histology slides.

Financial disclosure: None to report.

Informed consent was obtained for this case report.

## ABBREVIATIONS

CDC: CTLA-4 deficiency colitis
CHOP: cyclophosphamide, doxorubicin, vincristine, prednisolone
CMV: cytomegalovirus
CRP: C-reactive protein
CTLA-4: cytotoxic T-lymphocyte-associated protein 4
IBD: inflammatory bowel disease
ICI: immune checkpoint inhibitor
IV: intravenous
RR: reference range
TEff: effector T cells
TNF: tumour necrosis factor
TReg: regulatory T cells

## References

[1] Chandrasekaran P Krausz M Han Y The intestinal microbiome and metabolome discern disease severity in cytotoxic T-lymphocyte-associated protein 4 deficiency. Microbiome. 2025;13(1):51. 39934899 10.1186/s40168-025-02028-7 PMC11817180

[2] Westermann-Clark E Ballow M Walter JE. The new quest in CTLA-4 insufficiency: How to immune modulate effectively? J Allergy Clin Immunol. 2022;149(2):543–6. 34915039 10.1016/j.jaci.2021.11.020

[3] Schwab C Gabrysch A Olbrich P Phenotype, penetrance, and treatment of 133 cytotoxic T-lymphocyte antigen 4–insufficient subjects. J Allergy Clin Immunol. 2018;142(6):1932–46. 29729943 10.1016/j.jaci.2018.02.055 PMC6215742

[4] Uhlig HH Charbit‐Henrion F Kotlarz D Clinical genomics for the diagnosis of monogenic forms of inflammatory bowel disease. J Pediatr Gastroenterol Nutr. 2020;72(3):456–73. 10.1097/MPG.0000000000003017 PMC8221730 33346580

[5] Egg D Rump IC Mitsuiki N Therapeutic options for CTLA-4 insufficiency. J Allergy Clin Immunol. 2022;149(2):736–46. 34111452 10.1016/j.jaci.2021.04.039

[6] Schneider BJ Naidoo J Santomasso BD Management of immune-related adverse events in patients treated with immune checkpoint inhibitor therapy: ASCO guideline update. J Clin Oncol. 2021;39(36):4073–126. 34724392 10.1200/JCO.21.01440

[7] Hashash JG Francis FF Farraye FA. Diagnosis and management of immune checkpoint inhibitor colitis. Gastroenterol Hepatol (N.Y). 2021;17(8):358–66. 34602898 PMC8475264

[8] Navarini AA Hruz P Berger CT Vedolizumab as a successful treatment of CTLA-4–associated autoimmune enterocolitis. J Allergy Clin Immunol. 2017;139(3):1043–6.e5. 27908448 10.1016/j.jaci.2016.08.042

[9] Becker E Dedden M Gall C Residual homing of α4β7-expressing β1+PI16+ regulatory T cells with potent suppressive activity correlates with exposure-efficacy of vedolizumab. Gut. 2021;71(8):1551–66. 34462337 10.1136/gutjnl-2021-324868

[10] Card T Ungaro R Bhayat F Blake A Hantsbarger G Travis S. Vedolizumab use is not associated with increased malignancy incidence: GEMINI LTS study results and post‐marketing data. Aliment Pharmacol Ther. 2019;51(1):149–57. 31747086 10.1111/apt.15538 PMC7050439

[11] Pfeuffer S Nelke C Pawlitzki M Abatacept induces long-term reconstitution of the B-Cell niche in a patient with CTLA-4 haploinsufficiency. Neurol Neuroimmunol Neuroinflam. 2025;12(2):e200351. 10.1212/NXI.0000000000200351 PMC11655169 39689284

[12] Dhunputh C Ducassou S Fernandes H Abatacept is useful in autoimmune cytopenia with immunopathologic manifestations caused by CTLA-4 defects. Blood. 2022;139(2):300–4. 34714911 10.1182/blood.2021013496

[13] Tran NN Setty M Cham E Chan AY Ali S. CTLA‐4 haploinsufficiency presenting as extensive enteropathy in a patient with very early onset inflammatory bowel disease. JPGN Rep. 2021;2(3):e099. 37205940 10.1097/PG9.0000000000000099 PMC10191597

[14] Domènech E Vega R Ojanguren I Cytomegalovirus infection in ulcerative colitis: A prospective, comparative study on prevalence and diagnostic strategy. Inflamm Bowel Dis. 2008;14(10):1373–9. 18452205 10.1002/ibd.20498

[15] Lv YL Han FF Jia YJ Is cytomegalovirus infection related to inflammatory bowel disease, especially steroid-resistant inflammatory bowel disease? A meta-analysis. Infect Drug Resist. 2017;10:511–9. 29276397 10.2147/IDR.S149784 PMC5733908

[16] Wu XW Wu L Ji HZ Wang FY. Relationship between cytomegalovirus infection and steroid resistance in inflammatory bowel disease: A meta-analysis. Dig Dis Sci. 2015;60(11):3203–8. 26031424 10.1007/s10620-015-3733-6 PMC4621704

[17] Pillet S Pozzetto B Roblin X. Cytomegalovirus and ulcerative colitis: Place of antiviral therapy. World J Gastroenterol. 2016;22(6):2030–45. 26877608 10.3748/wjg.v22.i6.2030 PMC4726676

[18] Del Gaudio A Di Vincenzo F Petito V Focus on immune checkpoint inhibitors-related intestinal inflammation: From pathogenesis to therapeutical approach. Inflamm Bowel Dis. 2024;30(6):1018–31. 37801695 10.1093/ibd/izad229 PMC11144981

[19] Franklin C Rooms I Fiedler M Cytomegalovirus reactivation in patients with refractory checkpoint inhibitor-induced colitis. Eur J Cancer. 2017;86:248–56. 29055840 10.1016/j.ejca.2017.09.019

[20] Meserve J Aniwan S Koliani-Pace JL Retrospective analysis of safety of vedolizumab in patients with inflammatory bowel diseases. Clin Gastroenterol Hepatol. 2019;17(8):1533–40.e2. 30268561 10.1016/j.cgh.2018.09.035 PMC6594363

